# First Report of *Trichophyton indotineae* Infection in Hungary

**DOI:** 10.3390/jof11040248

**Published:** 2025-03-25

**Authors:** Zoltán Tóth, Beatrix Ványai, Renátó Kovács, Ágnes Jakab, Andrea Szegedi, Bence Balázs, László Majoros

**Affiliations:** 1 Department of Medical Microbiology, Faculty of Medicine, University of Debrecen, 4032 Debrecen, Hungary; 2 Medical Microbiology, Clinical Centre, University of Debrecen, 4032 Debrecen, Hungary; 3 Department of Dermatology and Venereology, Faculty of Medicine, University of Debrecen, 4032 Debrecen, Hungary; vanyai.beatrix@med.unideb.hu (B.V.);; 4 Dermatology Clinic, Clinical Centre, University of Debrecen, 4032 Debrecen, Hungary

**Keywords:** *T. indotineae*, susceptibility testing, sequencing

## Abstract

*Trichophyton indotineae* is associated with difficult-to-treat, often extensive dermatophytosis and resistance to the commonly used antifungal agents. Successful therapy often necessitates higher than usual doses of systemic therapy for prolonged periods. The spread of this species has gained much attention lately, as several European and other Western hemisphere countries have recently reported their first respective cases or increasing numbers of them. Until recently, this species was not described in Hungary. Here, we report a case caused by this species in a patient with a travel history to an endemic region. The isolate was identified preliminarily by MALDI-TOF mass spectrometry and confirmed by DNA sequencing; furthermore, it was subject to phenotypic antifungal susceptibility testing by broth microdilution to fluconazole, voriconazole, posaconazole, itraconazole, and terbinafine. According to the susceptibility results, the isolate was wild type to all tested agents, including terbinafine which was in line with the sequencing data, and with the uncommon excellent therapeutic response to topical allylamine treatment. This case also further confirms the applicability of the MSI-2 database for the rapid identification of *T. indotineae* in routine clinical microbiology laboratories as a cost-effective and simple method.

## 1. Introduction

*Trichophyton indotineae*, previously *T. mentagrophytes* genotype VIII, was recently separated from the *T. mentagrophytes* complex as an individual species based on its distinct mycological and clinical traits [1,2]. Considered as an anthropophilic species responsible for extensive tinea corporis and tinea cruris cases, this fungus is also associated with resistance to antifungal agents commonly administered for treating these infections, especially terbinafine [2,3,4]. In the recent years, *T. indotineae* overtook as the most prevalent *Trichophyton* species in India and became highly prevalent in other countries such as Iran or Bangladesh [2,5,6,7]. Nonetheless, the spread of this species is irrespective of geographical boundaries, and in the last years, several Western hemisphere countries have reported their first respective cases, including Italy, France, The United Kingdom, the United States, and Germany, among others [2,8,9,10,11,12,13]. While most of these are considered as imported, it is worrisome that cases without travel history to endemic regions and clear epidemiological linkage have been also described, suggestive of transmission already occurring in the community setting at previously non-endemic territories [10,11,12,13]. Identifying *T. indotineae* is challenging without complex molecular methods, as this species morphologically closely resembles other *T. mentagrophytes* complex genotypes [14]. Consequently, *ITS* (Internal Transcribed Spacer) sequencing is considered as a gold standard for species-level identification of *T. indotineae* [15]. Recently, the MSI-2 online fungal database was updated to be able to distinguish between *T. indotineae* and *T. mentagrophytes* based on the presence or absence of specific peaks in the mass spectrum, which may greatly simplify and speed up the identification procedure [4,16]. Here, we report the first described case of *T. indotineae* infection in Hungary, preliminarily identified by Matrix Assisted Laser Desorption/Ionization Time of Flight Mass Spectrometry (MALDI-TOF MS) with the MSI-2 database and confirmed by whole-genome sequencing (WGS).

## 2. Case Presentation

**Figure 1:**
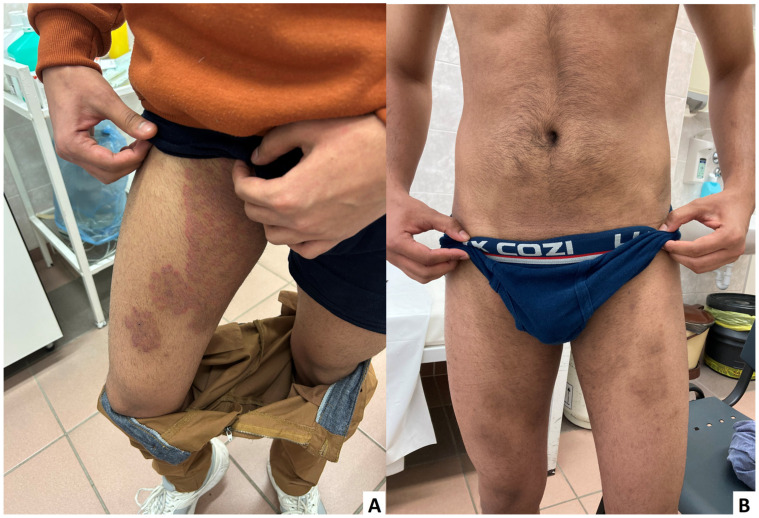
(**A**) Confluent patches seen on the first admission on the right thigh; (**B**) Almost completely healed lesions seen on the last visit of the patient after naftifine and vinegar treatment.

A male patient in his twenties was admitted to the Department of Dermatology and Venereology, Clinical Centre, University of Debrecen with reddish-brown fine scaly patches on both forearms and on the right thighs (Figure 1A), which were sometimes confluent. According to the patient, he did not use topical therapy and had no history of a notable disease prior. Based on the clinical picture, isoconazole-nitrate and diflurtocolone-valerate (10 mg/g and 1 mg/g, respectively) cream BID (twice daily), and fluconazole capsule (150 mg) twice weekly were recommended, and an appointment for control examination was arranged in three weeks. Mycological examination was not performed at that time. On revisit, he reported worsening symptoms with severe itching. On physical examination, on both forearms, and above the sternum, 5–10 cm large hyperaemic areas were observed, together with an area of scaly livid inflammation extending from the lower abdomen to the navel and the medial surface of the thighs and inguinal folds. Skin scraping was collected for mycological examination, and the recommended therapy was changed to daily washing with ketoconazole shampoo (20 mg/g) and application of terbinafine cream (10 mg/g) BID with a control examination in three weeks. The patient did not appear at the planned appointment, attending, however, three months later. On physical examination, lesions were almost completely healed, only post-inflammatory hyperpigmentation (Figure 1B) was observable, together with excoriated papules on the dorsal surface of the thighs. According to the patient, he was unsatisfied with the effectiveness of the prescribed regimen, and after three weeks he switched to naftifine ointment (10 mg/g) and vinegar (unknown concentration and frequency) topically as a home remedy for six weeks. He also mentioned that his sibling had similar symptoms on his visit home in India last year. Since the culture results were available at that time, and *T. indotineae* was confirmed with known susceptibility results, further therapy was switched to per os (itraconazole 100 mg) daily for at least four weeks. Since then, the patient has not attended any follow-ups. Written informed consent was obtained during his last visit.

## 3. Materials and Methods

### 3.1. Isolation and Culturing

Skin scrapings of the patient from the impacted site was submitted for culturing and identification to the Medical Microbiology, Clinical Centre, University of Debrecen on Sabouraud Dextrose Agar supplemented with chloramphenicol (Liofilchem, Roseto degli Abruzzi, Italy) and was incubated for eight days at room temperature before sufficient growth was achieved and MALDI-TOF analysis could be performed. The laboratory identifier assigned to the sample was 26337.

### 3.2. Identification by MALDI-TOF MS

An approximately 5 × 5 mm section of a colony was removed and placed in 500 µL 100% ethanol in a 1.5 mL centrifuge tube and was homogenized using a blunt toothpick. The homogenizate was centrfuged at 13,000 RCF for 5 min; then, the supernatant was removed and 100 µL of 70% formic acid was added. Following thorough vortexing, 1 µL was placed on the spots of an MSP96 target and was measured on a Bruker Biotyper Microflex LT instrument (Bruker Corporation, Billerica, MA, USA) using the NIH (National Institutes of Health) filamentous fungi spectrum acquisition protocol [17] and the Bruker Biotyper Filamentous Fungi library (version 4.0). Acquired spectra were uploaded to the MSI-2 database [16,18], which is routinely performed in our laboratory in case of *T. mentragrophytes* complex results are obtained with the Bruker Filamentous Fungi database, for rapid preliminary identfication of possible *T. indotineae* isolates. The mass peaks of the acquired spectra were annoted with Bruker FlexAnalysis (version 3.4).

### 3.3. Whole-Genome Sequencing (WGS)

For definitive identification of the isolate, WGS was performed. Genomic DNA was extracted using Zymo Quick-DNA Fecal/Soil (Zymo Research, Irvine, CA, USA) kit according to the manufacturer instructions regarding fungi. The library was prepared using ONT (Oxford Nanopore Technologies, Oxford, UK) Rapid Sequencing Kit V14 (SQK-RAD114) and was sequenced on an ONT MinION instrument on MIN114 flow cell. Reads shorter than 200 bp were rejected. Basecalling was performed by Dorado (version 7.3.9) embedded in the MinKNOW software (version 23.11.5) in super accurate mode (SUP). Since the first sequencing run did not yield enough reads, the same extracted nucleic acid was prepared again for a second run with the same library preparation kit. Reads that passed filtering by Dorado in default mode from both sequencing runs were concatenated by EPI2ME labs fastcat (version 0.15.1).

### 3.4. Whole-Genome Sequencing Data Analysis

Reads that passed filtering were mapped to the *ITS* (GenBank acc. number MW600653) [19], *SQLE* (Squalene–epoxidase, *ERG1*) (GenBank acc. number, MW187981) [20], and *ERG11b* (Lanosterol-14-demethylase) (GenBank acc. number MZ636375) [21] sequences using NextFlow wf-alignment algorithm (version 1.1.2) in default mode present in the EPI2ME lab software (version 5.1.10). Consensus sequence was extracted using Unipro Ugene (version 50.0) [22] from the bam files and multiple alignement was performed with CLUSTALW (version 2.1) [23] embedded in the Unipro Ugene software. The acquired *ITS* was compared to *T. indotineae* UCMS-IGIB-CI12, UCMS-IGIB-CI14 [19], CBS 146623 (ex-type strain), and *T. mentagrophytes* CBS 428.63 [1]. Single Nucleotied Polymorphisms (SNPs) were assessed manually.

### 3.5. Susceptibility Testing

Susceptibility testing was performed according to the EUCAST E.Def 11.0 protocol described by Arendrup et al. in RPMI-1640 glucose medium supplemented with cycloheximide and chloramphenicol [24]. Tested antifungals and concentration ranges were as follows: fluconazole (64–0.125 mg/L), voriconazole (4–0.008 mg/L), posaconazole (4–0.008 mg/L), itraconazole (4–0.008 mg/L), and terbinafine (4–0.008 mg/L). All the tested antifungal agents and media were obtained from Merck (Merck, Budapest, Hungary). The minimum inhibitory concentration (MIC) values were acquired using a Thermo-Fisher MiltiSKAN Sky microplate spectrophotometer at 490 nm (Thermo-Fisher Scientific, Waltham, MA, USA) after five days of incubation at room temperature. For quality control purposes, since no reference strain of *Trichophyton* sp. was available at the time, two *Candida* reference strains (*C. parapsilosis* ATCC 22019 and *C. krusei* ATCC 6258) were used [4]. The suscpetibility testing was performed twice on different days.

## 4. Results

### 4.1. MALDI-TOF MS

**Figure 2:**
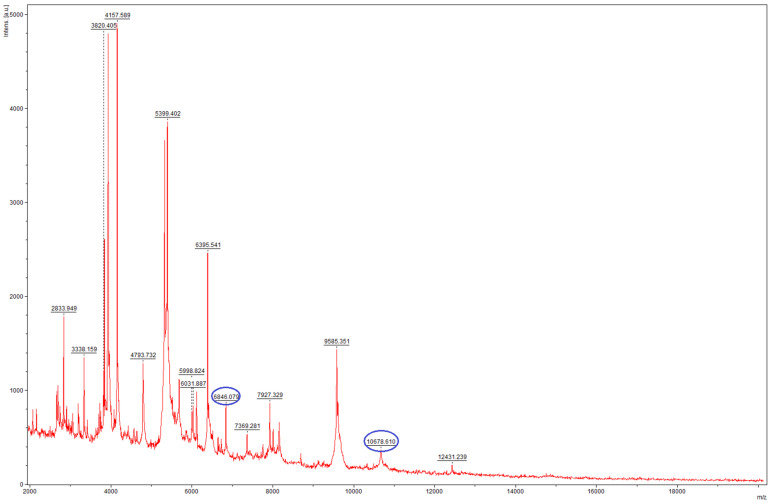
Mass spectrum of the isolate 26337 *T. indotineae*, acquired on a Bruker Microflex LT instrument. The typical peaks in the spectrum are highlighted in blue.

Bruker Compass (version 4.1) in Research Use Only mode identified the isolate as *T. mentagrophytes* complex, yet the score was below 2.00, which indicates that only genus level identification was possible with high confidence. MSI-2 identified the isolate as *T. indotineae*, with only B level evidence, however, which is practically similar to the level of confidence to genus-level identification by the Biotyper Compass. It should be noted that the Bruker Filamentous Fungi database does not include reference spectra for *T. indotineae* in the used version. On visual examination of the acquired spectra using Bruker FlexAnalyis (version 3.4), the two indicative peaks of *T. indotineae* at 6845 ± 5 Da and 10,680 ± 10 Da were detected (Figure 2) [16]. Based on these results, the isolate was deemed to be sequenced to confirm the MALDI-TOF MS identification results.

### 4.2. Whole-Genome Sequencing

**Figure 3:**
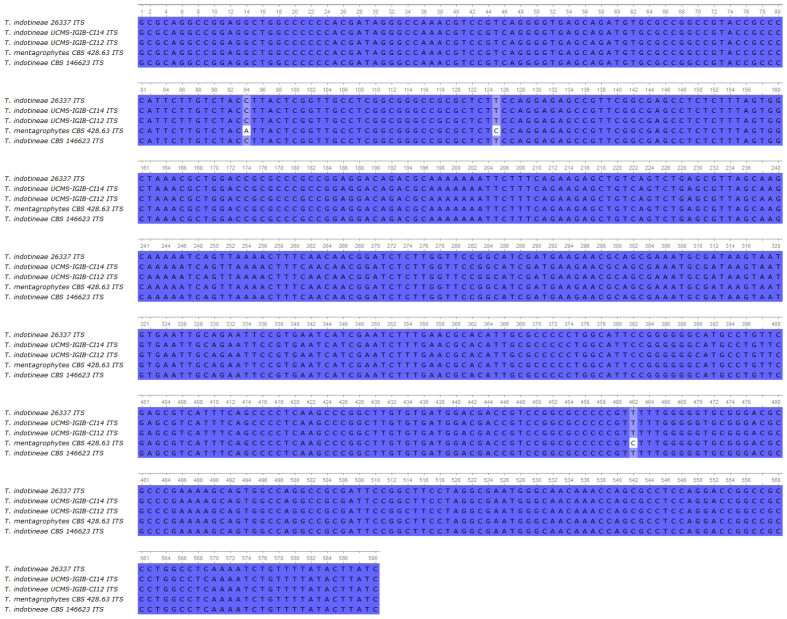
Multiple alignment of the ITS region of the isolate 26337 compared to UCMS-IGIB-CI14, UCMS-IGIB-CI12 *T. indotineae* strains [19], *T. mentagrophytes* CBS 428.63, and *T. indotineae* strain CBS 146623.

Sequencing data revealed that the isolate was indeed *T. indotineae*, showing 100% identity in of the ITS region compared to UCMS-IGIB-CI14, UCMS-IGIB-CI12, and CBS 146623 *T. indotineae* strains (Figure 3) [1,19]. In the *SQLE* gene, the isolate harbored a G1342A missense mutation, which resulted in an A448T amino acid change in the enzyme (Supplementary Figure S1). The *ERG11b* gene was found to be wild type compared to the sequence of the *T. indotineae* UKJ 476/21 (Supplementary Figure S2).

### 4.3. Susceptibility Testing Results

According to the susceptibility testing results, the isolate (26337) was highly susceptible to terbinafine (MIC 0.03–0.06 mg/L) and triazoles (posaconazole, voriconazole and itraconazole), except for the fluconazole, which had markedly higher MIC values (Table 1). The observed MICs were lower than the published tentative ECOFFs (Epidemiological Cut-Off Value) regarding itraconazole and terbinafine [25]. The MIC values of the *C. krusei* ATCC 6258 reference strain were within the accepted limits according to the EUCAST QC MIC values where available [26].

## 5. Discussion

Many of the described *T. indotineae* infections in the Western hemisphere are associated with international travel [8,9,10,11,12,13] and our presented case is not an exception. What is notable, however, is the unusually good therapeutic response with topical therapy, involving naftifine together with vinegar as a home remedy. Moreover, the presented case further supports the usefulness of MALDI-TOF MS for rapid and low-cost preliminary identification of *T. indotineae*. It should be also noted that this species had not been described in Hungary prior.

Recent literature recommends administration of systemic antifungals, mostly itraconazole alone or in combination with topical agents to treat *T. indotineae* infections [27,28,29] partly due to the alarmingly common resistance against terbinafine, reaching up to 70% [5]. Daily doses of 200 mg per os are generally administered with good efficacy; however, therapeutic failures with this regimen are also reported [2,28]. The length of itraconazole therapy varies widely between 1 and 12 weeks in the described cases [28], but at least 4–6 weeks of therapy is recommended [2]. Higher doses up to daily 400 mg have been also reported as safe and efficient, but the number of reports is considerably lower [28]. Increasing terbinafine resistance rates are also observed for other dermatophytes, for example, in Denmark, among other countries [30,31]. The mechanisms responsible for terbinafine resistance in *T. indotineae* are mostly missense mutations in the *SQLE* gene typically resulting in F397L or L393S amino acid substitutions [28]. The A448T substitution is also harbored by a considerable number of the *T. indotineae* isolates studied, but it does not result in terbinafine resistance if not present with other prominent mutations [28]. In our case, the isolate only had this latter amino acid alteration, which is in line with the low MIC values observed for terbinafine and may at least partially explain the good response to allylamine therapy, even though naftifine shows somewhat weaker in vitro activity compared to terbinafine against *Trichophyton* isolates [32]. Only one report is available where naftifine was used in combination with various agents against *T. indotineae*, ultimately resolved by oral itraconazole and topical clotrimazole after various regimens, but it should be noted that that isolate was terbinafine resistant in contrast to the presented case [27]. The role of topical vinegar in the resolution of the disease is unknown. While the main component acetic acid has potent antibacterial and antifungal effects [33], inappropriate application may result in skin damage [34]; therefore, it is not recommended for treating tinea corporis and cruris. On the other hand, it may have a role in treating fungal nail infections, via decreasing the pH locally, enhancing the activity of some topical antifungal preparations [35]. Notable exceptions are possibly allylamines, because their activity is actually decreased at lower pH [36,37].

The poor therapeutic response to per os fluconazole aligns with the literature [13,28,29], further suggesting that fluconazole has little role in treating *T. indotineae* infections. Efficacy data regarding isoconazole is lacking for *T. indotineae*. Considering its structural relatedness to miconazole [38] and comparable in vitro activity to other topical azoles, e.g., bifonazole [39], a similar efficacy could probably be expected. It should be noted, however, that the available in vitro activity data are fairly old, and it is unknown how well it applies to *T. indotineae*.

Isolates harboring A448T substitution in their squalene-epoxidase enzyme are associated with decreased azole susceptibility, yet not all the described isolates follow this pattern [21]. Ebert and colleagues observed for the first time that isolates carrying this mutation may show reduced azole sensitivity [5]. Yamada et al. found in their pioneering work that such isolates often have type II tandem repeat amplification of *ERG11b* region, resulting in significant target overexpression and increased itraconazole and voriconazole MIC values [40]. Nevertheless, such genomic amplification is not obligatory for significant overexpression and decreased itraconazole activity, suggesting further mechanisms may play a role [21,41]. The picture is toned by the results of De Paepe et al., who found that all the isolates they tested with A448T *SQLE* mutation had azole susceptibility patterns comparable to those with different mutations [4]. Notably, none of the isolates had higher itraconazole MICs than the tentative ECOFF defined by EUCAST [4]. Recently, Bhuiyan et al. found both itraconazole-susceptible and -resistant strains among A448T isolates [7]. The isolate in our study was also highly susceptible to azole-type agents, despite harbouring the A448T using EUCAST methodology. Unfortunately, we did not assess either the presence of tandem repeat amplification or the expression levels of *ERG11b*, which is a limitation of the present work, but the observed low azole MIC values make the presence of acquired resistance mechanisms somewhat unlikely in our opinion. Overall, it seems that the A448T does not define by itself whether an isolate has decreased azole susceptibility and further studies are needed to elucidate this issue.

While this is the first described *T. indotineae* infection in Hungary, this species may have been already present previously. The true extent of the distribution and frequency of *T. indotineae* may be underestimated globally [42], and the reason is likely multifaceted. One of them is that possibly not all the patients seek professional medical help and resort to home remedies and over-the-counter drugs. Even if they attend examinations, species-level identification or even culturing of dermatophytes is not necessarily pursued everywhere [43]; unfortunately, Hungary is no exception. While therapy-non-responding infections, as seen in our case, may attract the attention of the clinicians and warrant further investigation, that may not be the case for *T. indotineae* infections responding well to empirical antifungal therapy. Additionally, there is also a risk of publication bias towards resistant isolates [13]. All of this may possibly result in a lower reporting rate and cases may remain under the radar. While the use of MALDI-TOF MS could significantly simplify the otherwise complex identification processes [16], it still relies on the successful culturing of the isolate and does not address the issue of sensitivity, a well-known limitation [44]. Of note, commercial molecular methods, such as qPCR, could enhance sensitivity detecting dermatophytes [43,44,45]; there are currently no available commercial tests to identify *T. indotineae* at the species level, and their higher cost [44] may limit their utility to cases of recalcitrant infections. Until simple molecular methods become widely available and capable of distinguishing *T. indotineae* from other *T. mentagrophytes* genotypes, culturing combined with MALDI-TOF MS could serve as a practical, albeit imperfect, tool to monitor the epidemiology and distribution of *T. indotineae*. However, laboratories should first make dermatophyte culture widely available, and clinicians should request it at least in suspected cases.

## Figures and Tables

**Table 1:** MIC values of the tested antifungal agents in mg/L, against *T. indotineae* isolate 26337, *C. krusei* ATCC6258 and *C. parapsilosis* ATCC22019.

Antifungal	*T. indotineae* 26337	*C. krusei* ATCC6258	*C. parapsilosis* ATCC22019
Fluconazole	16	16–32	0.25
Voriconazole	0.06–0.12	0.06	0.016
Posaconazole	0.008	0.03	0.016
Itraconazole	0.008	0.03	0.03
Terbinafine	0.03–0.06	4	0.25–0.5

## Data Availability

ITS, partial SQLE and partial ERG11b sequences are available under the following accession numbers: PQ836183, PQ999201 and PQ999202. The Whole Genome Shotgun project is available at NCBI BioProject under accession number PRJNA1224191 with SRA accession numbers SRR32343822 and SRR32343823.

## Supplementary Materials

The following supporting information can be downloaded at: https://www.mdpi.com/article/10.3390/jof11040248/s1, Figure S1: Multiple alignment of the partial *SQLE* gene of the isolate *T. indotineae* 26337 compared to VCPI 1005/P/17, 211509/17, I5, *T. indotineae* strains [20] and *T. mentagrophytes* XM11; Figure S2: Alignment of the partial ERG11b gene of the isolate *T. indotineae* 26337 compared to *T. mentagrophytes* (*T. indotineae*) strain UKJ 476/21 [21].
