# Supplementary material for: First Report of Trichophyton indotineae Infection in Hungary
Source: J Fungi (Basel). 2025 Mar 25;11(4):248. doi: 10.3390/jof11040248 (PMC12028727; doi:10.3390/jof11040248)

Supplementary Figure S2. Alignment of the partial *ERG11b* gene of the isolate *T. indotineae* 26337 compared to *T. mentagrophytes* (*T. indotineae)* strain UKJ 476/21 [21]

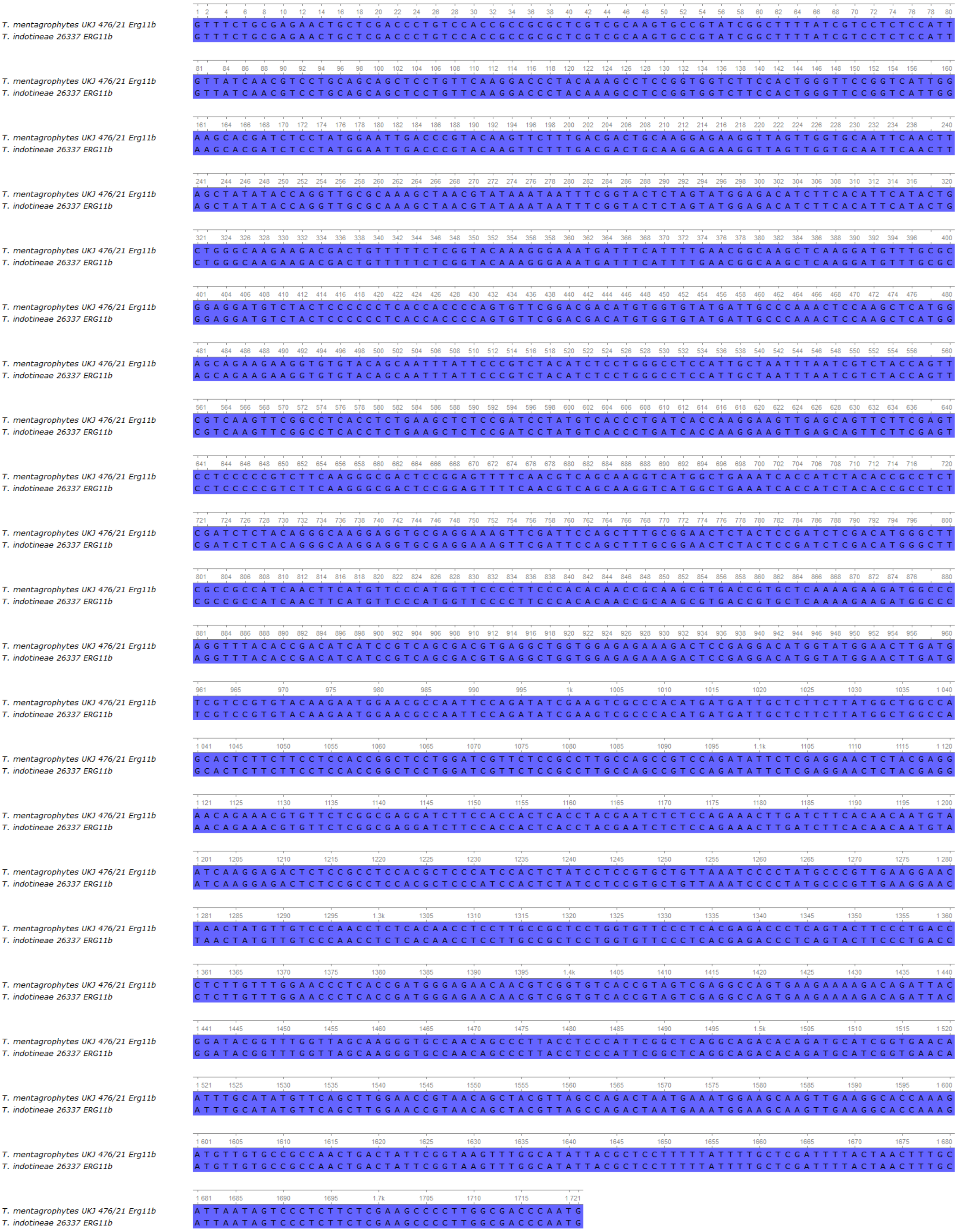

Supplement: Supplementary file 1 [file jof-11-00248-s001.zip › Figure S2.pdf]
