# Supplementary material for: First Report of Trichophyton indotineae Infection in Hungary
Source: J Fungi (Basel). 2025 Mar 25;11(4):248. doi: 10.3390/jof11040248 (PMC12028727; doi:10.3390/jof11040248)

Supplementary Figure S1. Multiple alignment of the partial *SQLE* gene of the isolate *T. indotineae* 26337 compared to VCPI 1005/P/17, 211509/17, I5, *T. indotineae* strains [20] and *T. mentagrophytes* XM11

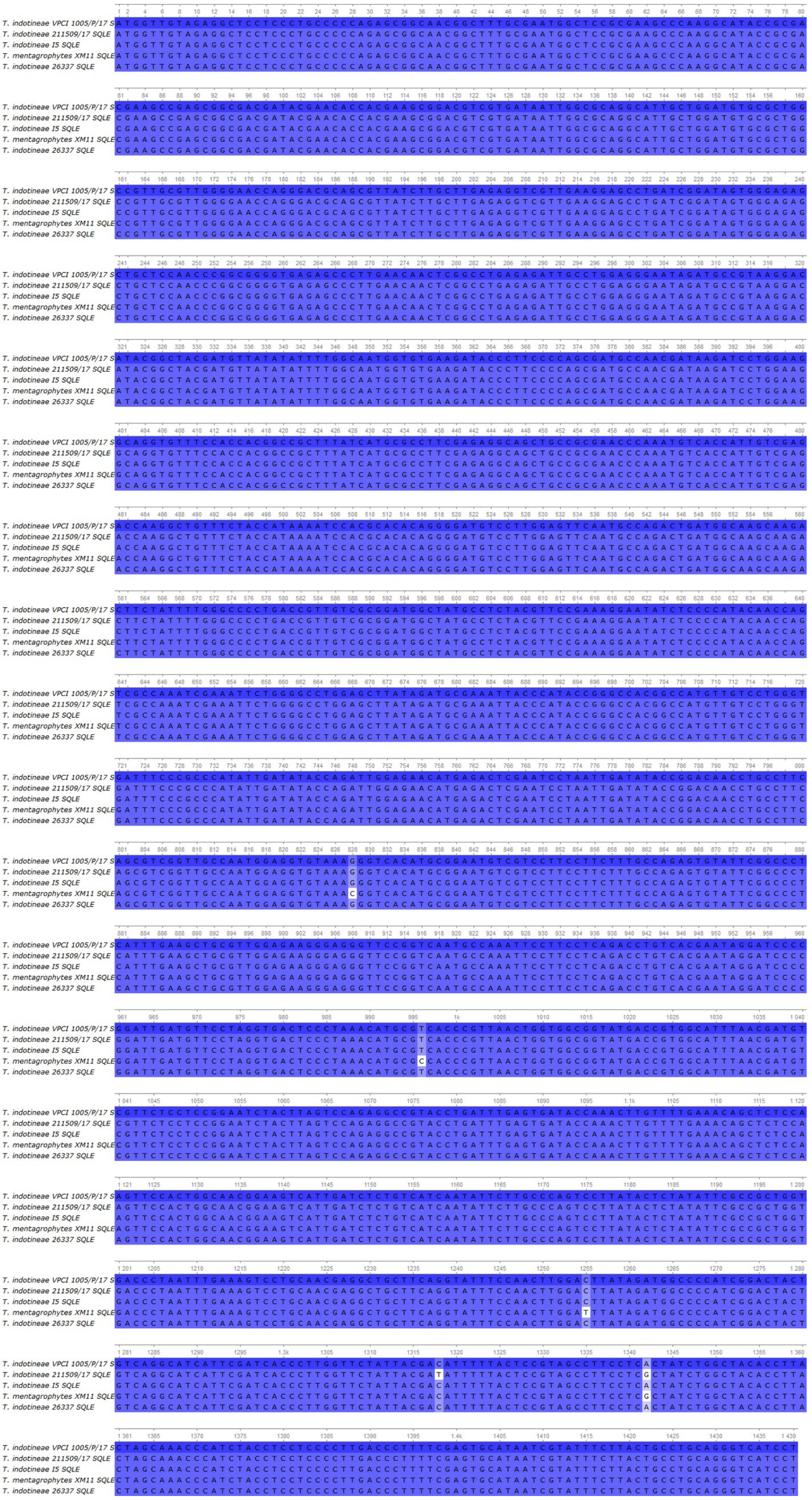

Supplement: Supplementary file 1 [file Figure S1.pdf › Figure S1.pdf]
